# Privacy-preserving record linkage in large databases using secure multiparty computation

**DOI:** 10.1186/s12920-018-0400-8

**Published:** 2018-10-11

**Authors:** Peeter Laud, Alisa Pankova

**Affiliations:** 1 Cybernetica AS, Ülikooli 2, Tartu, 51003 Estonia; 2 STACC, Ülikooli 2, Tartu, 51003 Estonia

**Keywords:** Secure multiparty computation, Privacy-preserving record linkage, Deduplication, Privacy

## Abstract

**Background:**

Practical applications for data analysis may require combining multiple databases belonging to different owners, such as health centers. The analysis should be performed without violating privacy of neither the centers themselves, nor the patients whose records these centers store. To avoid biased analysis results, it may be important to remove duplicate records among the centers, so that each patient’s data would be taken into account only once. This task is very closely related to privacy-preserving record linkage.

**Methods:**

This paper presents a solution to privacy-preserving deduplication among records of several databases using secure multiparty computation. It is build upon one of the fastest practical secure multiparty computation platforms, called Sharemind.

**Results:**

The tests on ca 10 million records of simulated databases with 1000 health centers of 10000 records each show that the computation is feasible in practice. The expected running time of the experiment is ca. 30 min for computing servers connected over 100 Mbit/s WAN, the expected error of the results is 2^−40^, and no errors have been detected for the particular test set that we used for our benchmarks.

**Conclusions:**

The solution is ready for practical use. It has well-defined security properties, implied by the properties of Sharemind platform. The solution assumes that exact matching of records is required, and a possible future research would be extending it to approximate matching.

## Background

In this paper, we present a solution to the first track of iDASH 2017 competition [1], titled *De-duplication for Global Alliance for Genomics and Health (GA4GH)*. The goal is to develop privacy-preserving record linkage (PPRL) technique on top of existing European ENCCA Unified Patient Identifier (EUPID) framework to facilitate the deduplication task in GA4GH [2].

There is a large number of data providers, called *healthcare centers*. They all have lists of patients. They want to know whether anyone else has the same patient in the list. This knowledge would allow them to exclude the duplicates from further data analysis, making it more efficient and less biased. Since each center uses its own local identities for its patients, they need to use so-called *quasi-identifiers* such as patient name and his date of birth to link the records, and this information may be sensitive. The centers could potentially agree on some kind of anonymization, replacing the quasi-identifiers with some random numbers. However, this is in general not sufficient, as e.g. seeing that some patient is present in exactly the centers A, B, C could already potentially leak more information about the patient. For example, if the observer already knew the patient records of A, he would in addition learn that these patients have also visited the centers B and C. Mechanisms stronger than anonymization are needed to overcome this problem.

### Technical details of the task

In the competition setting, the patient list of a health center is presented as a table, the columns of which are correspond to the patient’s sensitive data, such as “first name”, “last name”, “date of birth”, “sex”, etc. Two records are considered the same if a certain combination of their attributes matches, e.g. concatenation of the name and the date of birth. For simplicity, it is assumed that the records are already pre-formatted, and possible typos should not be taken into account (we will discuss exact and approximate matching in the “Discussion” section). For each record, the centers compute the required combination of the attributes, and hash it. They then upload all hashes to the ideal computing machine, implemented with the help of Secure Multiparty Computation (SMC). The machine tells to each center, which of its records are also present in someone else’s dataset, but it does not tell to whom exactly these records belong. The machine itself should be realized by *k*≥3 specially assigned servers, and no server should learn the values of uploaded hashes, since hashing is used only for shortness of representation and does not provide privacy by itself.

The targeted size of the computation is ca. 1000 centers, with ca. 10000 records each. The competition organizers have made available a test set with 1000 tables, each containing around 10000 records.

There are certain details about the data input and output, which make this task more specific:

- The data is not uploaded instantaneously by all centers. It is uploaded over time, so the ideal computing machine may have significant resources left over, while waiting for the next inputs.
- The first uploader of each record does not get it indicated as a duplicate, even if more copies of the same record are uploaded later. The record is indicated as a duplicate only to the next uploaders.

**Fig. 1:**
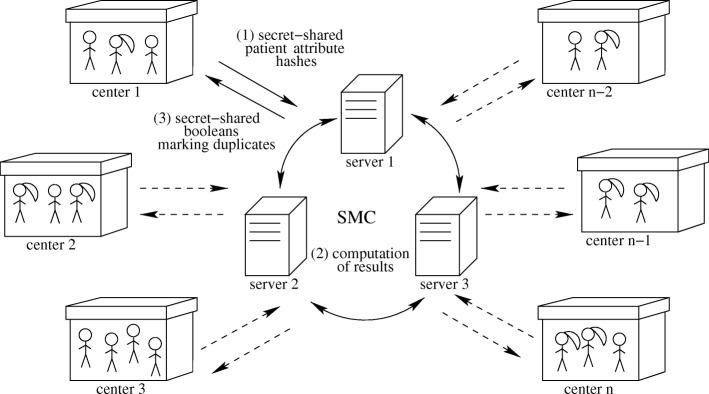
System diagram. The setting to which the solutions proposed in this paper are applied

The system diagram is depicted in Fig. 1. There are *n* healthcare centers, indexed in the order they provide data. The data is uploaded to the servers in form of secret-shared hashes of records (1). After SMC servers compute the results on this data (2), they report to each center a list of booleans marking the duplicates (3), i.e. the records that have already occured in the centers with smaller indices. The results are returned to the centers in a secret-shared manner, so that no server will learn them, but the receiver center is able to reconstruct his answer from shares. In the end, each center knows which records it should discard to avoid repetitions with the other centers, and the remaining records may now be used for any other task, e.g. data analysis using other SMC protocols.

There can be two ways in which the final outputs should actually be delivered to the centers. These are different tasks with different challenges and solution possibilities. We will consider them both.

1. In our first solution, the results are sent out to the centers only after all uploads have finished. It is fine as far as all 1000 centers will agree to contribute their data more or less simultaneously, so that the first uploader will not have to wait for too long. We provide a faster and a more secure algorithm for this setting.
2. In our second solution, each upload of a bunch of records by a center is immediately followed by a response from the servers. This algorithm has to be used in the case when some centers may need more time to contribute their data, but the faster uploaders already want to get the results. It is more difficult to preserve privacy in this case.

### Related work

There are numerous papers about PPRL, including (but not limited to) solutions based on Bloom filters for string approximate matching [3, 4], extensions of Bloom filters to matching of numerical data [5], as well as provably secure multiparty protocols [6]. Some practical implementations of PPRL include [7], describing a protocol where the linking is delegated to the patient himself, and [8], based on matching seeded hashes, which describes in details which preprocessing steps should be applied to the data to make it better linkable. An extensive state-of-the art overview of this topic in the beginning of 2017 has been given in [9].

Similarly to other privacy-preserving computation tasks, the two main approaches to PPRL are Secure Multiparty Computation (SMC) techniques and data perturbation techniques. The SMC approach is in general less efficient, but it provides clear security guarantees, while data perturbation methods can be severely broken due to informal security definitions that they provide [10]. For example, some cryptanalysis of Bloom filter based solutions has been performed in [11, 12]. They show some interesting attacks, including patient name frequency analysis. The iDASH 2017 competition has been focused on SMC methods.

SMC often uses the notion of three separate roles for parties [13]. There are *input parties* that provide the computation with data, which needs protection. There are *computation parties* that execute the privacy-preserving computation on provided data. The computation parties should not learn anything new from the execution, besides what is leaked through the design of the application. Finally, there are *result parties* who learn the results of the computation. These three sets of parties may intersect. In the context of the competition task, the computation parties are specially assigned servers, whose number does not depend on the total number of health centers participating. The centers themselves are both input and result parties, each of them receiving a different subset of the outcomes.

In our solution, we assume three computation parties, since this is the setting for which the fastest SMC protocols are known, and mature computation platforms exist. The solution makes use of the following assumptions, allowed by the competition:

1. Honest majority: at most 1 of 3 servers can be corrupted.
2. Honest-but-curious (passive) adversaries: the corrupted server follows the protocol, but the attacker may get access to all messages received by this server.

As far as these assumptions are satisfied, the only things that may be inferred by the servers from the computation are the following. The first type of leakage is the “duplication pattern”: *for each* $n\in \mathbb {N}$, *how many records are there, that occur in the lists of all data providers exactly n times?* In the context of our task, this leaks how many patients have been registered in exactly *n* hospitals, but this number is not correlated with particular records or hospitals in any way. The second type of leakage is: *how many duplicates have been reported to the health center?* It does not allow to correlate the duplicates of different centers in any way, and it only leaks that some *n* patients of this center have visited some other center. We note that, in our solutions, either the first or the second type of leakage takes place, but never both at once.

Some points for evaluating a PPRL algorithm have been mentioned in [9]. According to their framework, our solution could be assessed as follows:

- **Scalability:** The network communication complexity of the first algorithm is *O*(*n*), where *n* is the total number of records that all the clients hold. e.g. if there are *m* clients holding *k* records each, then *n*=*mk*. The complexity of the second algorithm is formally *O*(*n*^2^), but we keep the constant small by using an elaborated filtering technique, which allow to reduce the number of comparisons up to 10 times compared to straightforward pairwise comparison, while slightly sacrificing the answer precision. We note that the running time of the second solution is actually more affected by heavy local computation than by network communication.
- **Linkage quality:** Our solution aims to achieve exact matching, as it was required by the test dataset. The loss in correctness is limited by 2^−40^ in our application. It happens mainly due to the initial application of a hash function to the inputs, which was a competition’s requirement anyway, and also due to dropping some bits in order to match data types that Sharemind supports. In the second solution, some more correctness is sacrificed to gain better efficiency.
- **Privacy:** It is guaranteed that we leak no more than the total number of patients that have been registered in exactly *n* hospitals (the first solution), or the number of duplicated records in one client’s data (the second solution).

## Methods

### The Sharemind platform

Our SMC protocols are implemented on top of the Sharemind^®;^ platform [14]. The platform provides a distributed virtual machine (VM) that must be installed at each of the computing servers. The machine interprets the description of a privacy-preserving application (in our case, the deduplication), where the cryptographic details are abstracted away. From the application developer’s point of view, different pieces of data are merely labeled as “public” or “private”. The private data will never be learned by any of the servers, unless it is deliberately *declassified* (i.e. opened to all servers) by the application. While the public values are stored at each server in plain, the private values are stored in a secret-shared manner, preventing a single server from learning its value. Underneath, the virtual machine executes cryptographic protocols to perform operations on private secret-shared data, which in general requires interaction between the servers. The underlying cryptographic protocols have been proven to be composable [15], meaning that the applications do not need to undergo any additional security proofs. Only deliberate declassification of values needs to be justified. This also concerns the deduplication that we present in this paper.

The main protocol set of Sharemind [16], denoted shared3p, is based on additive sharing among three parties. The private representation of a value *u*∈*R* from a finite *ringR* is denoted by *⟦u⟧*, and is defined as *⟦u⟧*=(*⟦u⟧*_1_,*⟦u⟧*_2_,*⟦u⟧*_3_), where the *share* *⟦u⟧*_*i*_∈*R* is held by the *i*-th server. The shares are random elements of *R*, with the constraint that the sum up to *u*. Hence, if anyone manages to get only one or two shares of *u*, he cannot infer any information about *u*. In [16], the authors have presented protocols for a number of basic arithmetic, relational and logical operations, such as addition, multiplication, comparisons, etc., transforming shares of the inputs into shares of the output. The supported rings are $\mathbb {Z}_{2^{n}}$ and $\mathbb {Z}_{2}^{n}$, and several different rings may be in used simultaneously in the same application. There are special protocols for converting shares between different rings. This basic set of operations has been extended in numerous follow-up papers [17–22].

The protocols of shared3p are secure against one passively corrupted party. Our deduplication application is built on top of them. In the rest of this section, we present all algorithms that we used in our application. In the loops, we use **foreach** and **while** to denote parallelizable loops, whilst **for** loops are sequential. Parallelization is important due to the latency of all non-linear operations with private values, due to the need to exchange messages between computation servers. We denote vectors as $\vec {x} = \langle {x_{1},\ldots,x_{n}}\rangle $.

### The first solution: output all results once in the end

In out first solution, the results of computation are only available at the end, after all *clients* (the health centers) have uploaded the hashes of their records. The outline of the process is the following. In the first phase, the SMC servers are collecting input data from the clients, without performing any deduplication detection on it. When the uploads have ceased (e.g. the deadline or some other trigger event has happened), the servers stop collecting the data. They run the deduplication algorithm on all data they managed to collect so far, and give to each client its personal output. If a record is duplicated, then the client that first uploaded it will not be notified that it is a duplicate. All other clients that have uploaded the same record will receive a notification about it.

#### Cryptographic building blocks

We fix two cryptographic functions: the hash function *H*:{0,1}^∗^→{0,1}^*η*^, and a block cipher $E:\{0,1\}^{\eta }\times \mathcal {K}\rightarrow \{0,1\}^{\eta }$, where $\mathcal {K}$ is the set of possible keys for *E*. The block size of *E* should be the same as the output length of *H*, so that we could apply *E* directly to the output of *H*. The challenge is that we will apply *E* to secret-shared values, and hence the block cipher *E* has to be easily computable under SMC. We have picked AES-128 as *E*, and the privacy-preserving implementation of AES-128 is already available in Sharemind [19]. There exist newer, possibly more efficient implementations of AES [23], as well as proposals for SMC-optimized block ciphers [24], which have not been implemented on Sharemind yet, and could potentially speed the computation up.

In our solution, we have taken *η*=128. We let *H* be the composition of SHA-256 cryptographic hash function and a universal one-way hash function family (UOWHF) [25].


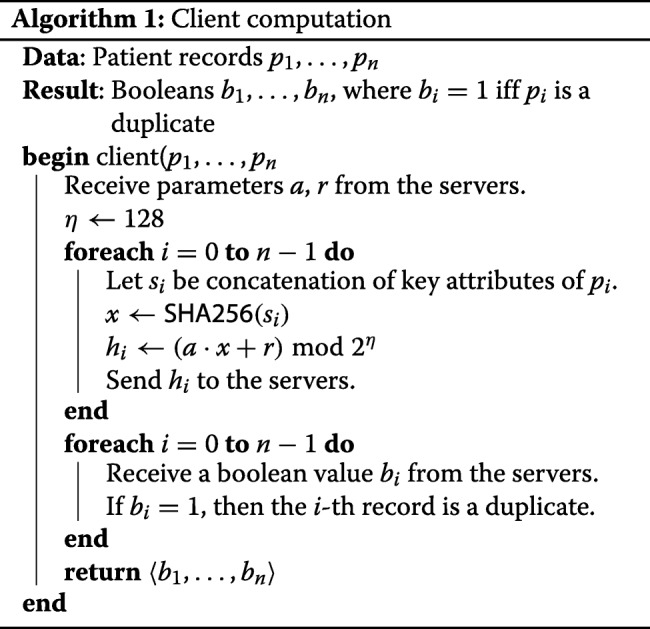


#### Computation on the client side

The behaviour of a client is described in Algorithm 1. At start-up, each client queries the Sharemind servers for the random parameters of the UOWHF *H*, which the servers generate in the beginning. Each client takes the input records as soon as they become available, picks the necessary attributes from each record, applies *H* to them, and uploads the hashes to Sharemind servers in secret-shared manner. The Sharemind API readily supports that. At this point, no server learns the exact values of the hashes, since they are all secret-shared.

In the end, when the servers have finished the computation, the client queries them for its personal result. The servers respond with the shares of the output, which is a vector of booleans of the same length as the number of records from this client, indicating whether a record is a duplicate one. The client reconstructs the result vector. Again, the Sharemind API already has support for that.

#### Computation on the server side

We describe the work of the servers in phases.

##### Start-up.

This short phase is given in Algorithm 2. The servers privately generate the following random values:

- parameters of the hash function *H*;
- a key *⟦K⟧* for the block cipher *E*.

All these values are generated in such a way that they remain secret-shared among the servers, and no server actually learns them. Sharemind API supports such shared randomness generation, and we denote the corresponding functionality as *random*.

The servers initialize a public variable *cnt* ←0. It will be used for indexation of clients.


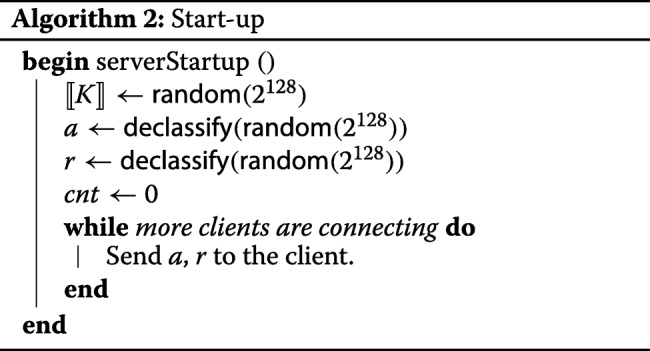


##### Upload

During the upload phase, two different activity threads are essentially taking place in parallel.

One thread is the actual acceptance of data from the clients, described in Algorithm 3. The hashes of clients are stored into an private array *⟦v⟧*, and the corresponding client identities into a public array *s* under the same indices. In principle, this algorithm could be invoked several times for the same client, if it intends to split up the upload of its data. Several clients may want to connect to the servers at the same time, so we need to make use of Sharemind’s database support to avoid race condition on *cnt*.


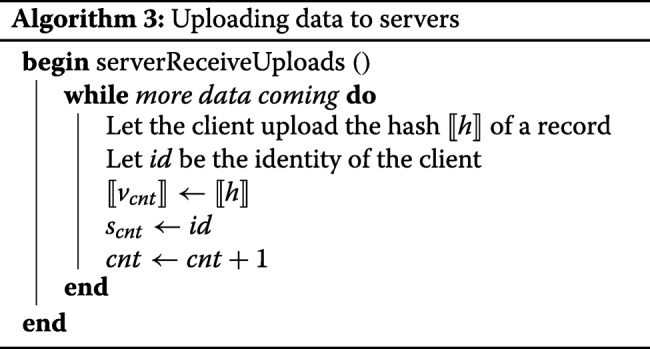


The other thread of activity is encrypting the elements *⟦v*_*i*_*⟧*. For each *i*, the servers evaluate *E* on *⟦v*_*i*_*⟧* in a privacy-preserving way, using the same key *⟦K⟧* for each encryption, and take the first 64 bits of the result, as described in Algorithm 4. The only reason why we take only 64 of 128 bits is that the largest ring that Sharemind currently supports is $\mathbb {Z}_{2^{64}}$. There are no problems with taking only 64 bits, as long as there are no collisions. With the envisioned amounts of data (around 10^7^=2^23.5^ records in total), collisions are unlikely; the birthday paradox puts their probability at around 2^−64+2·23.5^=2^−17^. We note that the communication complexity also reduces with the number of bits of the data types.


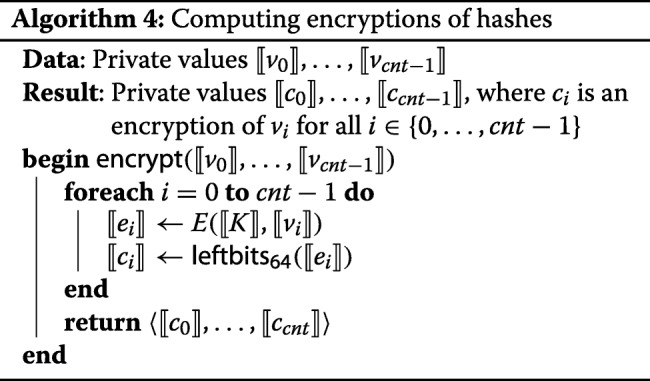


In our implementation, evaluation of *E* can take place either immediately after the data upload, or it can be run in a separate thread, waiting until more data comes to process it all in parallel. In any case, the application of *E* is done in parallelized manner, batching up the hashes that are waiting for being processed so far.

##### Computation of results.

When the upload has finished and the ciphertext *⟦c*_*i*_*⟧* has been computed for each *i*∈{0,…,*cnt*−1}, then the final results are computed as in Algorithm 5. First of all, all computed ciphertexts are privately shuffled, and then declassified. Since they are encrypted with a block cipher whose key remains secretshared, it only reveals the “duplication pattern”, i.e. how many values occur there exactly *n* times. The private shuffle [26] in the shared3p protocol set is highly efficient: the amount of communication is linear in the size of the shuffled vector, and the number of communication rounds is constant. It also allows to easily apply the shuffle inverse, which is as efficient as the shuffle itself.


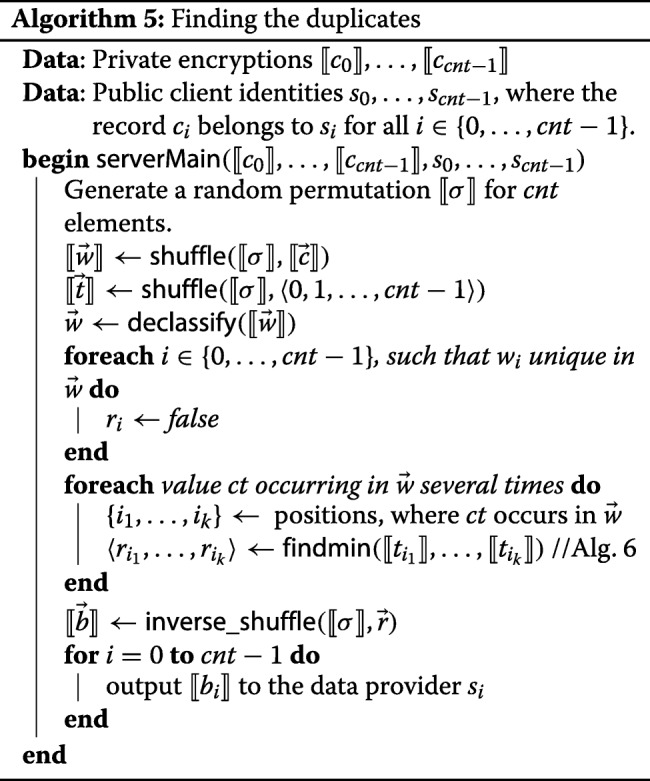


At this point, the servers could already mark the duplicates with boolean values 1 and the remaining values with 0. After the obtained vector is privately shuffled back, the servers can return the secret shared bits to the clients, so that the *i*-th clients learns the bits indicating the duplicity of its own records. The problem is that the servers need to notify all clients except the first one, but because of shuffling they do not know which entry belongs to the first client. They cannot declassify the client indices *⟦s*_*i*_*⟧* either since it would partially undo the shuffling.

In order to determine which value belongs to the first client, the servers run Algorithm 6. This algorithm is applied to each set of shuffled entries that have identical values, to determine the minimum amongst them. The minimum should be labeled *false* since it is not considered a duplicate, and all other elements should be labeled *true*. The idea behind this recursive algorithm is the following. The inputs are split into pairs, and a comparison performed within each pair. The element that is larger is definitely not the minimum of the entire set, so we can immediately write *b*_*i*_:=*true*. The indices of all elements that turned out to be smaller are stored into *m*_*i*_, and the whole algorithm is now applied again to the elements indexed by *m*_*i*_. The procedure is repeated until there are is only one element left, which is the minimum, so the algorithm returns *false* in one-element case.

Although Algorithm 6 works with private values, it reveals the ordering between certain elements of $\vec {t}$. Because of the random shuffle, the ordering of this vector is completely random, so it does not disclose any information. No server learns from the output more than it would from a random permutation.


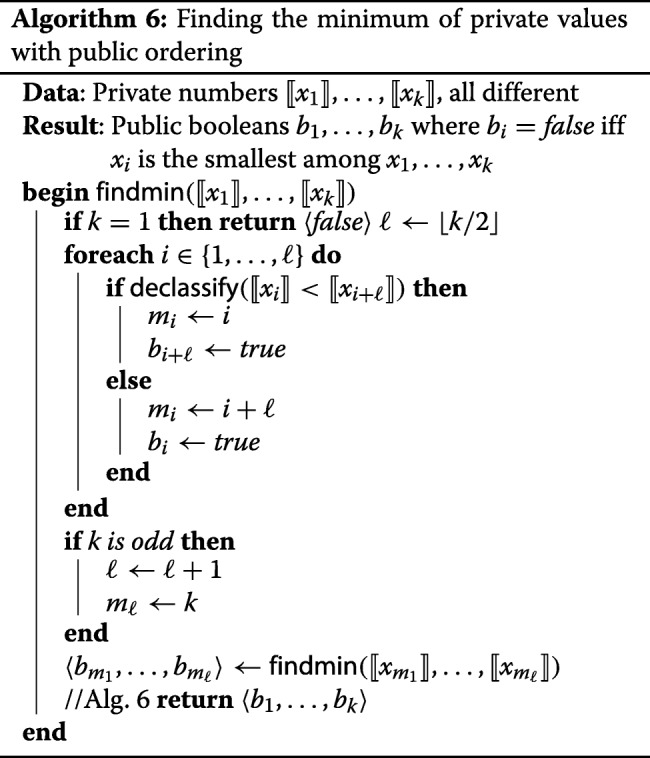


### The second solution: output results immediately

Our second solution considers the case where the client is immediately notified, which of its hashes of records have already been uploaded by some previous client. This task is more difficult than the previous one. We cannot simply declassify the encrypted hashes when they arrive, since it would leak which pairs of centers have intersecting sets of records.

The second solution has the same components *E* and *H* as the first solution, constructed in the same manner. Again, the computation servers agree on the key *⟦K⟧* in the beginning, as well as the parameters of *H*.

#### Computation on the client side

The clients perform pretty much in the same manner as in the first solution, according to Algorithm 1. The only technical difference is that the servers react to the upload immediately, so the client most likely does not interrupt the session with the servers, and stays connected until it receives the result.

#### Computation on the server side

Assume that the servers already store *T* encrypted hashes (zero or more), provided by the previous clients. These *T* hashes have been stored as *public* values *z*_1_,…,*z*_*T*_, where *z*_*i*_ is equal to the first 64 bits of *E*(*K*,*v*_*i*_). Duplicates have already been removed among *z*_1_,…,*z*_*T*_. Assuming that duplicates never end up among *z*_1_,…,*z*_*T*_, since *⟦K⟧* remains private, *z*_1_,…,*z*_*T*_ are computationally indistinguishable from *T* uniformly randomly distributed values, so it is safe to make them public.

The 64-bit values *z*_1_,…,*z*_*T*_ are kept in 2^*B*^*buckets*, numbered from 0 to 2^*B*^−1. In our implementation, *B*=16. Each bucket is just a vector of values. Each *z*_*i*_ is assigned to the bucket **B**_*j*_, where *j* is equal to the first 16 bits of *z*_*i*_.

Now suppose that a center has uploaded the hashes *⟦v*_1_*⟧*,…,*⟦v*_*t*_*⟧*. The servers need to check whether these hashes has occurred before. The computation starts by encrypting each *⟦v*_*i*_*⟧*. Let $\llbracket {z^{\prime }_{1}}\rrbracket,\ldots,\llbracket {z^{\prime }_{t}}\rrbracket $ be the results of encryption, computed by $\llbracket {z^{\prime }_{i}}\rrbracket =\mathsf {{leftbits}_{64}}(E(\llbracket {K}\rrbracket,\llbracket {v_{i}}\rrbracket))$. We cannot immediately declassify them since it would leak which pairs of centers have intersecting sets of records. Instead, we should use privacy-preserving comparison.

We do not want to simply invoke the private comparison protocol for each *z*_*i*_ and $\llbracket {z^{\prime }_{j}}\rrbracket $, because we consider their number to be too large. Indeed, as we are aiming to handle ca. 10 million records, this method would cause us to compare each pair of records, leading to ca. 5·10^13^ invocations of the comparison protocol. An *ℓ*-bit comparison requires slightly more network communication than a *ℓ*-bit multiplication [16], with the latter requiring each computation server to send and receive two *ℓ*-bit values [17]. If *ℓ*=64 and there are 5·10^13^ operations, then each server has to send out and receive at least 6·10^15^ bits, which on a 100 Mb/s network (specified in the conditions of the competition task) would take almost two years.

We reduce the number of comparisons in the following manner. Let *⟦z*^′^*⟧* be one of the private values $\llbracket {z^{\prime }_{1}}\rrbracket,\ldots,\llbracket {z^{\prime }_{t}}\rrbracket $; all *t* values are handled in parallel. The comparison of *⟦z*^′^*⟧* with *z*_1_,…,*z*_*T*_ is described in Algorithm 7. In this algorithm, we let *N* be the maximum size of a bucket. We denote the *j*-th element of the *i*-th bucket by **B**_*i*,*j*_. We assume that each bucket has exactly *N* elements, adding special dummy elements if necessary.

The *characteristic vector* of an element $x\in \mathbb {Z}_{M}$ is a vector $\langle {b_{0},\ldots,b_{M-1}}\rangle \in \mathbb {Z}_{2}^{M}$, where *b*_*x*_=1 and all other elements are equal to 0. The shared3p protocol set has a simple and efficient protocol for computing characteristic vectors, described in [27]. The protocol turns a private value into a private characteristic vector. The characteristic vector of *leftbits*_16_(*⟦z*^′^*⟧*) marks the index of the bucket to which *⟦z*^′^*⟧* belongs, and the expression $\bigoplus _{i=0}^{2^{B}-1}\llbracket {b_{i}}\rrbracket \cdot \mathbf {B}_{i,j}$ returns exactly the *j*-th element of that bucket, which we denote *⟦y*_*j*_*⟧*. This way, the values *⟦y*_1_*⟧*,…,*⟦y*_*N*_*⟧* are the privately represented content of the bucket, into which *z*^′^ would belong. The private comparison $\llbracket {z^{\prime }}\rrbracket \stackrel {?}{=}\llbracket {y_{j}}\rrbracket $ is performed for all *j*, thus comparing *⟦z*^′^*⟧* against each element that belongs to the *i*-th bucket. Finally, $\llbracket {b}\rrbracket =\bigvee _{j=1}^{n}\llbracket {c_{j}}\rrbracket $ is the private OR of all comparisons, which tells whether there had been at least one match. The private bits *⟦b*_1_*⟧*,…,*⟦b*_*t*_*⟧* resulting from applying Algorithm 7 to all $\llbracket {z^{\prime }_{1}}\rrbracket,\ldots,\llbracket {z^{\prime }_{t}}\rrbracket $ are returned to the client.


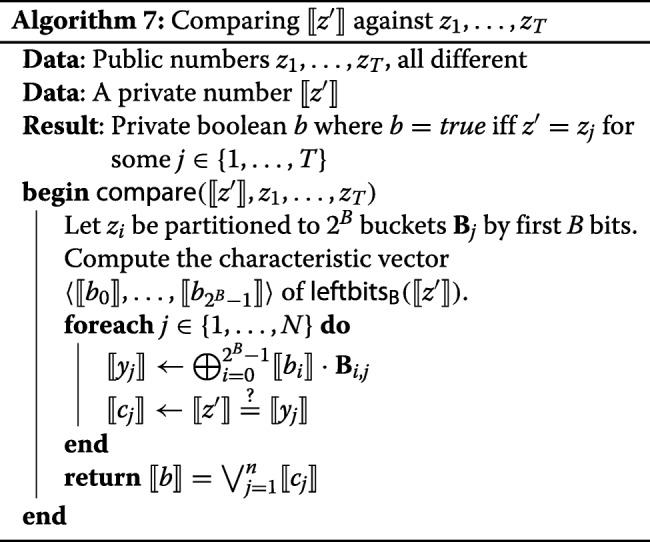


After returning the answer to the client, the buckets have to be updated with $z^{\prime }_{1},\ldots,z^{\prime }_{t}$. It is safe to declassify $\llbracket {z^{\prime }_{i}}\rrbracket $ if *b*_*i*_=0, since in this case $\llbracket {z^{\prime }_{i}}\rrbracket $ cannot be correlated to any of *z*_*i*_ and is indistinguishable from a random value. However, we cannot immediately declassify the vector $\llbracket {\vec {b}}\rrbracket $, because the positions of duplicated elements may give away information about the input data. Since we are allowed to leak the total number of duplicated entries per client, we can do as described in Algorithm 8:

1. randomly shuffle 〈*⟦b*_1_*⟧*,…,*⟦b*_*t*_*⟧*〉 and $\langle {\llbracket {z^{\prime }_{1}}\rrbracket,\ldots,\llbracket {z^{\prime }_{t}}\rrbracket }\rangle $, using the same permutation;
2. declassify $\llbracket {\vec {b}}\rrbracket $;
3. declassify those *⟦zi*′*⟧*, where *b*_*i*_=0, and add these $z^{\prime }_{i}$ to the respective buckets.

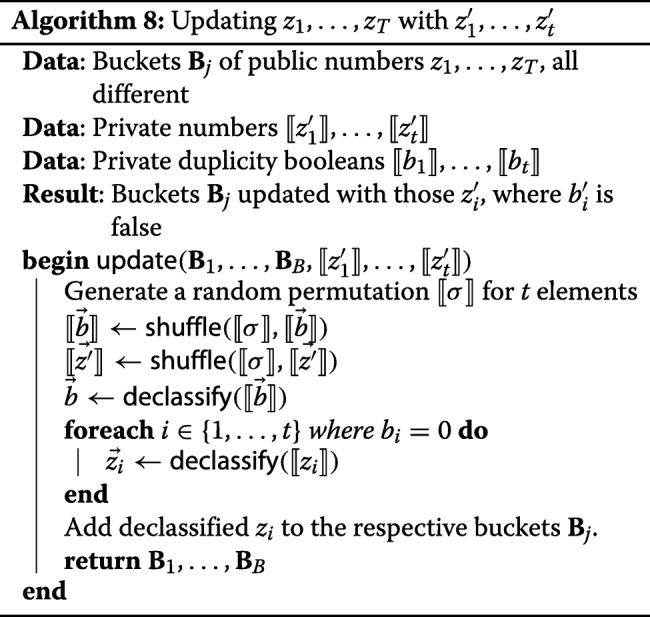


In our implementation, *⟦z*^′^*⟧* is shared over $\mathbb {Z}_{2}^{64}$, hence taking the first 16 bits of it is a local operation, resulting in a value in $\mathbb {Z}_{2}^{16}$. The characteristic vector protocol in [27] is easily adaptable to compute the characteristic vectors of elements of $\mathbb {Z}_{2}^{B}$, and its result is a vector over $\mathbb {Z}_{2}$ with length 2^*B*^. The computation of the characteristic vector requires communication of two elements of $\mathbb {Z}_{2}^{B}$ and one element of $\mathbb {Z}_{2}^{2^{B}}$ (in *total*, not per party).

The computation of *⟦y*_*j*_*⟧* in Algorithm 7 is again a local operation. The computations of *⟦c*_*j*_*⟧* and their disjunction are straightforward using the protocols available in the shared3p set of Sharemind.

#### Speeding up local computations

In Alg. 7, the computation of *⟦y*_*j*_*⟧* is local. Nevertheless, in practice it takes a major part of the entire effort of the protocol, taking up most of the time in it. If we knew something about the size of *z*^′^, we could compute $\bigoplus $ not over all buckets, but only over a subset of them, covering only the range into which *z*^′^ is guaranteed to fall, with a negligible error.

A bucket is defined by the most significant bits of elements that it contains. If we take any two buckets, then all elements in one of them will be strictly smaller than all elements in the other one. If we sort $\llbracket {z^{\prime }_{1}}\rrbracket,\ldots,\llbracket {z^{\prime }_{t}}\rrbracket $ in ascending order (Sharemind has efficient protocols for sorting private values [28]), we know that the first elements more likely belong to the buckets with “smaller” bits, and the last elements more likely belong to buckets with “larger” bits. We can estimate these probabilities more precisely.

As the key *⟦K⟧* is secret, and the hashes *⟦v*_1_*⟧*,…,*⟦v*_*t*_*⟧* are all different, the values $\llbracket {z^{\prime }_{1}}\rrbracket,\ldots,\llbracket {z^{\prime }_{t}}\rrbracket $ can be treated as mutually independent, uniformly random elements of $\mathbb {Z}_{2}^{64}$. After sorting them, their likely ranges can be derived from the order statistics as follows.

Let $\mathcal {P}$ be a discrete probability distribution over values *x*_1_,*x*_2_,…, such that the probability mass of *x*_*i*_ is *p*_*i*_. Let *X*_1_,…,*X*_*n*_ be random variables sampled from $\mathcal {P}$, and let $X^{\prime }_{1},\ldots,X^{\prime }_{n}$ be obtained after sorting *X*_1_,…,*X*_*n*_ in ascending order. We have $\text {Pr}[X^{\prime }_{j} \leq x_{i}] = \sum _{k=j}^{n}{{n}\choose {k}}P_{i}^{k} \cdot (1 - P_{i})^{n-k}$, where $P_{i} = Pr[X_{i} \leq x_{i}] = \sum _{k=1}^{i} p_{k}$. This quantity comes from summing up probabilities of all possible combinations, where at least *j* of *n* variables are smaller than *x*_*i*_. For a fixed *j*, this expression is actually the cumulative density function of a binomial distribution *B*(*n*,*P*_*j*_).

In our case, $\mathcal {P}$ is the distribution over AES ciphertexts, i.e *p*_*i*_=2^−128^ for all *i*. The sorted ciphertexts $z^{\prime }_{i}$ are instances of random variables $X^{\prime }_{i}$. We want to find *m*_*i*_ and *M*_*i*_, such that $\text {Pr}[z^{\prime }_{i}< m_{i}]\leq \varepsilon $ and $\text {Pr}[z^{\prime }_{i}>M_{i}]\leq \varepsilon $, where *ε* is the desired error probability. Since we are dealing with binomial distribution, we can use e.g. Höffding’s inequality

$$\text{Pr}[z^{\prime}_{i} \leq m_{i}] \leq exp\left(-2\frac{(n \cdot P_{i} - m_{i})^{2}}{n}\right)$$

and Chernoff’s inequality

$$\text{Pr}[z^{\prime}_{i} \leq m_{i}] \leq exp\left(-\frac{1}{2P_{i}}\cdot\frac{(n\cdot P_{i}-m_{i})^{2}}{n}\right)\enspace,$$

where *exp*(*x*)=*e*^*x*^ for Euler’s number *e*. We can solve the equation $\epsilon = exp(-2\frac {(n \cdot P_{i} - m_{i})^{2}}{n})$ if $P_{i} \leq \frac {1}{4}$, and $\epsilon = exp(-\frac {1}{2P_{i}}\frac {(n \cdot P_{i} - m_{i})^{2}}{n})$ if $P_{i} \geq \frac {1}{4}$, getting the value for *m*_*i*_. The value for *M*_*i*_ can be obtained analogously, since $\text {Pr}[z^{\prime }_{i} \geq x_{i}] = \text {Pr}[-z^{\prime }_{i} \leq -x_{i}]$.

By default, we use *ε*=2^−40^ as the probability of error. As the total number of hashes is expected to be around 1000·10000≈2^23.5^ and we have two bounds to try for each hash, the probability of making a bounds check error during the whole run is not more than 2·2^−40+23.5^=2^−15.5^, which we consider acceptable, and which is also similar to errors due to the collisions in the first 64 bits of AES output.

The usefulness of these bounds increases together with *t*. If *t*=100 (and *ε*=2^−40^), then we gain little, as the ranges [*m*_*i*_,*M*_*i*_] still cover around half of the whole range. If *t*=10000, then the sorted values can be much more tightly positioned — the ranges [*m*_*i*_,*M*_*i*_] are less than 1/10 of the whole range.

#### Alternative comparison

The communication costs of Algorithm 7 may be further reduced, ultimately turning them into a constant (assuming that *B* is constant), albeit with a further increase in the costs of local computation.

Consider the bucket **B**_*i*_ with elements **B**_*i*,1_,…,**B**_*i*,*N*_. The value *z*^′^ is an element **B**_*i*_ iff it is a root of the polynomial $\mathbf {P}_{i}(x)=\prod _{j=1}^{N}(x-\mathbf {B}_{i,j})$. The polynomial is considered over the field $\mathbb {F}_{2^{64}}$. The elements of this field are 64-bit strings and their addition is bitwise exclusive or. Hence, an additive sharing over $\mathbb {F}_{2^{64}}$ is at the same time also sharing over $\mathbb {Z}_{2}^{64}$ and vice versa.

Let **P**_*i*,0_,…,**P**_*i*,*N*_ be the coefficients of the polynomial **P**_*i*_. It does not make sense to compute **P**_*i*_(*⟦z*^′^*⟧*) in a straightforward way, because this would involve *N*−1 private multiplications for each bucket. A better way is given in Algorithm 9.


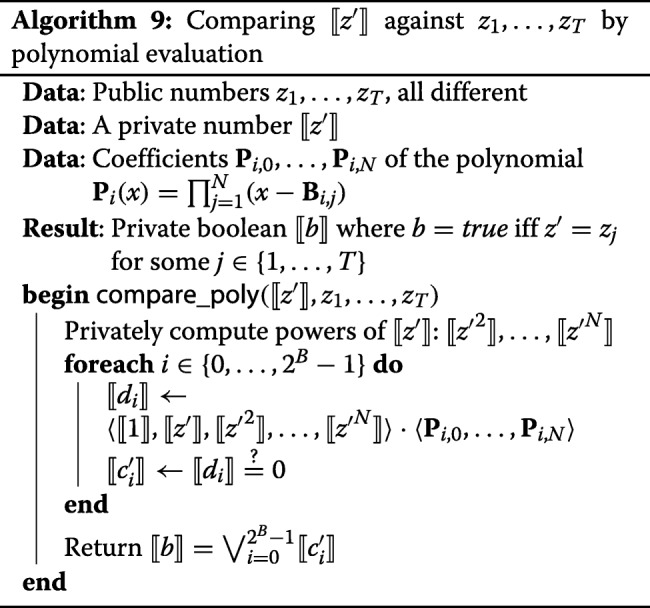


In Algorithm 9, *⟦d*_*i*_*⟧* is computed as a scalar product of the private vector of the powers of *z*^′^ with the public vector of the coefficients of **P**_*i*_. The powers of *⟦z*^′^*⟧* have to be computed only once for all buckets. These powers are computed with the help of the usual multiplication protocol of Sharemind, but working over $\mathbb {F}_{2^{64}}$. The local operations in this protocol are multiplications in $\mathbb {F}_{2^{64}}$, which are relatively more expensive than ordinary bitwise operations. In our implementation we use the NTL library [29] for binary field operations. The computation of all *⟦d*_*i*_*⟧* involves many multiplications in this field, so even though the computation does not require any communication between the parties, it is quite heavy on the local side.

As the computation of *⟦z*^′^^2^*⟧*,…,*⟦z*^′^^*N*^*⟧* is done over a field, we can push its heaviest part to the *precomputation phase*, leaving just a single private multiplication to be done during runtime. The method is described in ([30], Algorithm 1). The precomputation consists of generating a random invertible element $\llbracket {r}\rrbracket \in \mathbb {F}_{2^{64}}^{*}$ together with its inverse *⟦r*^−1^*⟧* and computing *⟦r*^2^*⟧*,…,*⟦r*^*N*^*⟧*. During the runtime, one computes *⟦z*^′^*⟧*·*⟦r*^−1^*⟧* and declassifies it. For an exponent *k*, the private value *⟦z*^′^^*k*^*⟧* is then found as *⟦z*^′^^*k*^*⟧*=(*z*^′^·*r*^−1^)^*k*^·*⟦r*^*k*^*⟧*, which can be computed locally, without interaction.

In binary fields, including $\mathbb {F}_{2^{64}}$, squaring of additively shared values does not require communication between the servers. This can be used to speed up precomputations. In ([30], Algorithm 4) it is shown how to reduce the *communication* cost of computing *⟦r*^2^*⟧*,…,*⟦r*^*N*^*⟧* to that of approximately $\sqrt {N}$ multiplications.

The polynomial-based comparison method is also amenable to the order statistic related speedup described in the previous section. Both comparison methods have been implemented in our second solution.

## Results

We have evaluated our solution on the test data provided by iDASH 2017 competition organizers. The data consists of 1000 CSV formatted files, ca. 10000 rows each. The evaluation has been performed on a local cluster of three 2 x Intel Xeon E5-2640 v3 2.6 GHz/8GT/20M servers, where one server has also played the role of all clients. The network bandwidth has been throttled to 100 Mb/s, and latency changed to 40 ms. The number of computation threads was limited to 2, as required by the competition rules. The data upload times have not been measured since they depend on the network connection between the server and the centers, and are not related to the particular SMC algorithms.

The number of incorrect answers in our benchmarks was 0, and it turns out that the error 2^−40^ was fine for 1000 clients with 10000 records each.

### First solution

The benchmarks of our first solution are given in Table 1. The entire computation takes ca. 32 min, after which all centers receive back the list of entries that are duplicates. This is quite fast for privacy-preserving computation with such large inputs, and in practice setting up the servers and collecting the data may even take more time than the computation itself.

**Table 1:** First solution

Encrypting the hashes	29 m
Shuffling the encrypted hashes	60 s
Find the first element in each duplicated set	30 s
Unshuffling the boolean results	12 s
Public sort, housekeeping	18 s
**Total**	32 m

### Second solution

**Fig. 2:**
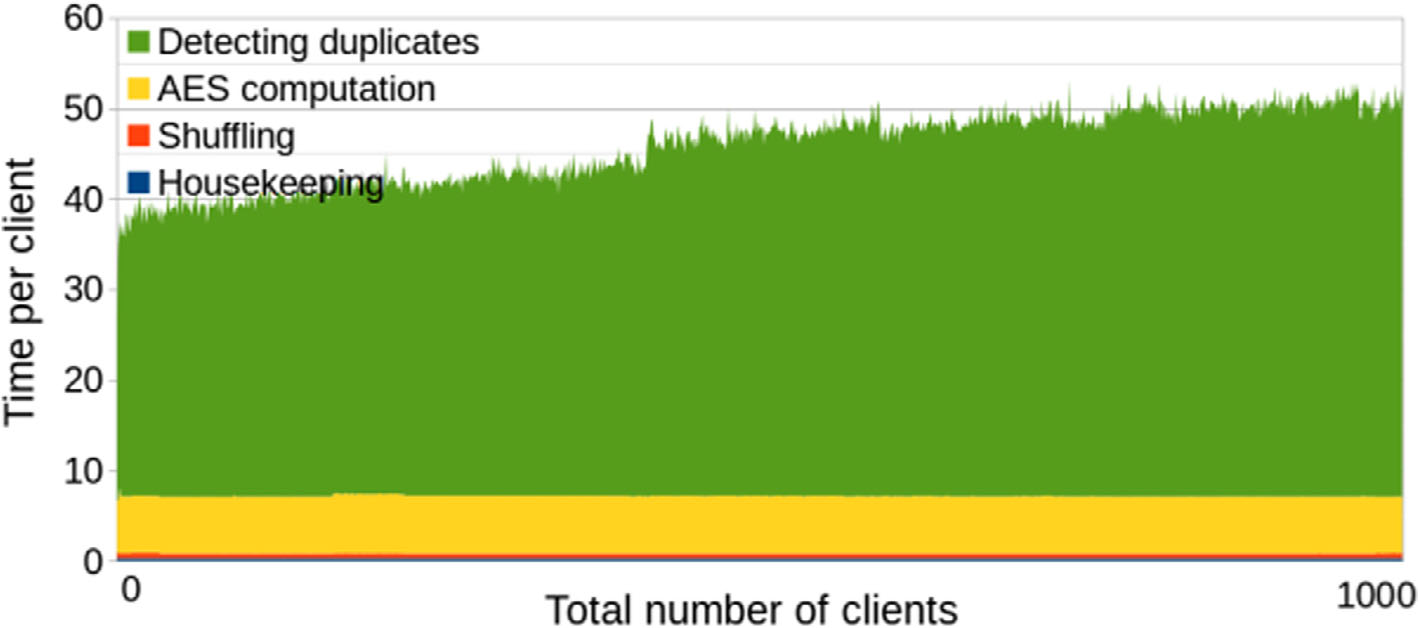
Second solution (w/o polynomials). Efficiency graph of the second solution without polynomial optimization. The number of CPU threads is limited to 2

If the centers do not provide their data simultaneously, and the time span between the first and the last contribution is long, then the first uploaders may probably not want to wait for the remaining ones. The 32 min of the actual computation may become insignificant compared to the time waiting for the other centers, so even a slower algorithm may give advantage if it returns the results immediately. The benchmarks of our second solution (*without* polynomial optimization) are given in Fig. 2 and summarized in Table 2, and they show that the waiting time for each server is between 20 and 45 s. The later the center joins, the longer will be his computation time. For all 1000 centers altogether, the second solution would give 12 h 45 min compared to the 32 min of the first solution. However, if the time span between the first and the last contributions exceeds at least 12 h 15 min, then the second method already gives advantage also in the total time.

**Table 2:** Second solution (w/o polynomials)

Housekeeping (avg per client)	0.28 s
Encrypting the hashes (avg per client)	6.3s
Shuffling the encrypted hashes (avg per client)	0.51 s
Detecting duplicates (min of 1000 clients)	20s
Detecting duplicates (max of 1000 clients)	46s
**Total** (1000 clients)	12 h 45 m

**Fig. 3:**
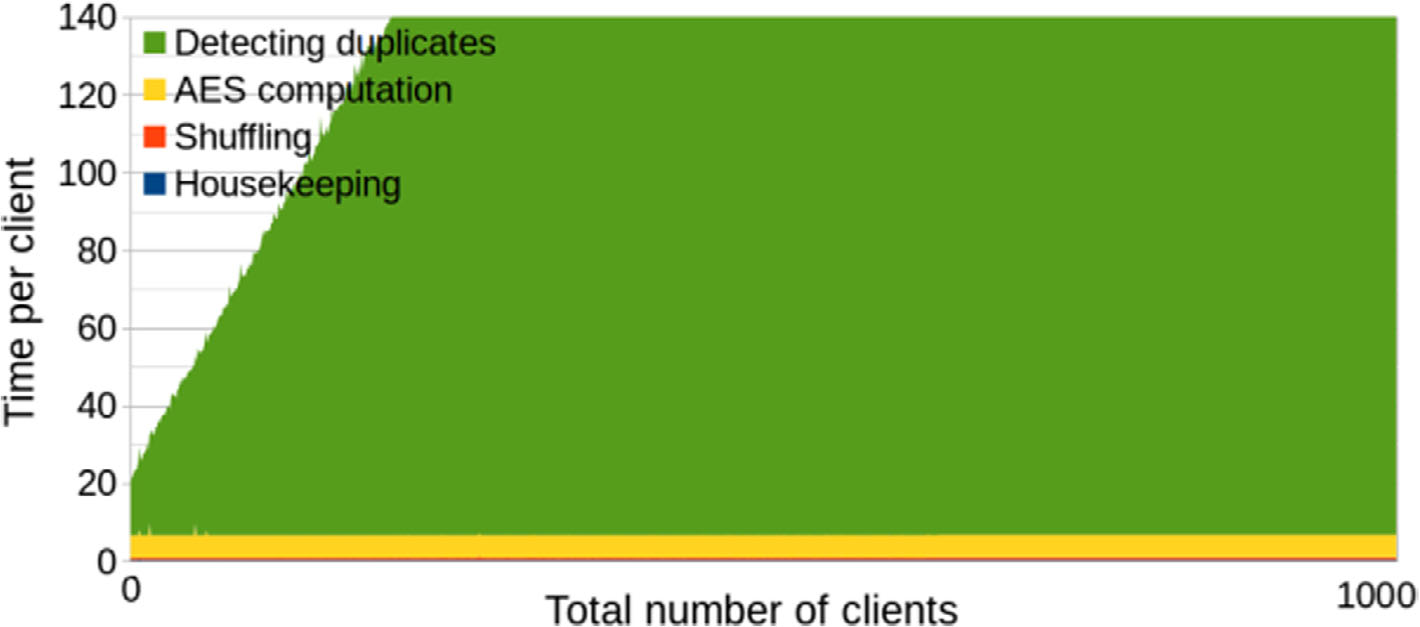
Second solution (w/ polynomials). Efficiency graph of the second solution with polynomial optimization. The number of CPU threads is limited to 2

The benchmarks of the second solution *with* polynomial optimization are given in Fig. 3 (the limit on Y-axis values is set to 140 to make it comparable to the other graphs) and summarized in Table 3. We see that, although the communication between parties decreases, the computation is slower. The time cost per client visibly grows linearly with the number of clients, so the total time is quadratic in the number of clients. This means that the multiplication in $\mathbb {F}_{2^{64}}$ is too heavy, and even limiting network connection to 100Mbps and adding 40ms latency did not make its complexity less significant. The total time of processing 1000 clients was almost seven times slower than the solution without polynomials. Nevertheless, for small number of clients, this solution is actually more efficient, and total time is smaller for the first 55 clients. The time *per client* has started getting worse after the 30-th client, so it is reasonable to switch between comparison algorithms at this point. Since the alternative comparison has less network communication, it should be theoretically better in settings with very slow network connection and fast hardware. We decided to conduct one more experiment to verify this claim.

**Table 3:** Second solution (w/ polynomials)

Housekeeping (avg per client)	0.28 s
Encrypting the hashes (avg per client)	5.8 s
Shuffling the encrypted hashes (avg per client)	0.53 s
Detecting duplicates (min of 1000 clients)	14 s
Detecting duplicates (max of 1000 clients)	666 s
Total (1000 clients)	88 h 8 m

**Fig. 4:**
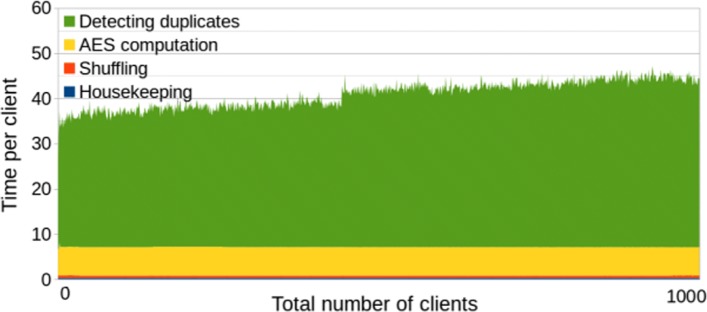
Second solution (w/o polynomials, no thread limit). Efficiency graph of the second solution without polynomial optimization. The number of CPU threads is 32

**Fig. 5:**
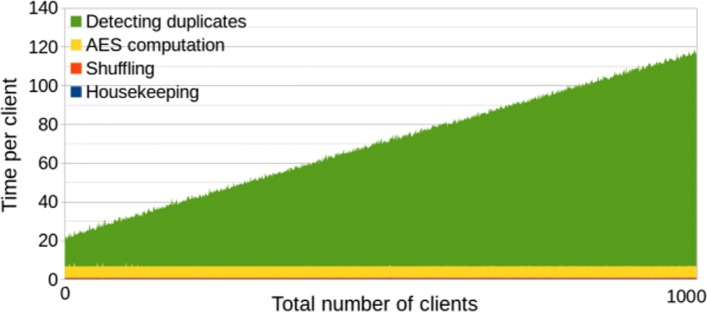
Second solution (w/ polynomials, no thread limit). Efficiency graph of the second solution with polynomial optimization. The number of CPU threads is 32

During the competition, the solutions were evaluated using only 2 parallel processor threads, but we have also tried to run the same algorithm without limiting their number (using all 32 cores), to see if we get any advantage. The results of the benchmarks with and without polynomials can be found in Figs. 4 and 5, summarized in Tables 4 and 5 respectively. We see that the polynomial solution was still slower, although all 32 processors on all three servers worked with 100% load during the main computation. However, while the time improvement of the solution without polynomials is not too significant (although 1.5 extra hours still make a difference), we see that the performance of polynomial solution is much better than it was with 2 threads, and the 1000 clients have been processed in 19 h 30 m instead of 88 h 8 m. This time, the polynomial solution was more efficient that the non-polynomial solution with up to 300 clients. The time *per client* has started getting worse after the 160-th client. We see that the alternative comparison indeed scales better w.r.t. parallelization.

**Table 4:** Second solution (w/o polynomials, no thread limit)

Housekeeping (avg per client)	0.28 s
Encrypting the hashes (avg per client)	6.3 s
Shuffling the encrypted hashes (avg per client)	0.50 s
Detecting duplicates (min of 1000 clients)	20 s
Detecting duplicates (max of 1000 clients)	40 s
**Total** (1000 clients)	11 h 20 m

**Table 5:** Second solution (w/ polynomials, no thread limit)

Housekeeping (avg per client)	0.28 s
Encrypting the hashes (avg per client)	5.8 s
Shuffling the encrypted hashes (avg per client)	0.55 s
Detecting duplicates (min of 1000 clients)	14 s
Detecting duplicates (max of 1000 clients)	112 s
**Total** (1000 clients)	19 h 30 m

## Discussion

We think that the results are good enough and can be applied to real data. Depending on the requirements, either the first or the second solution can be used.

The data that was provided by iDASH 2017 competition organizers was idealized, i.e. it was already preprocessed and assumed that e.g. the gender is labeled exactly “M” and “F” and there are no other possible encodings. In a practical application, we should think how much preprocessing we agree to delegate to the data owner, and how much we perform using secure multiparty computation.

We note that revealing “duplication pattern” is safe as far as there are sufficiently many clients, so that leaking how many patients have been present in exactly *n* centers would be safe. This leakage would be bad in some extreme cases, e.g with two medical centers with one patient, since then the server will know whether that particular patient has been in both centers or only one of them. However, if the data is small, then slower and more secure algorithms could be used for that anyway, such as finding privacy-preserving set intersection between each pair of medical centers to determine the duplicates.

One possible problem of Sharemind deployment is that its protocols work under assumptions of the honest majority and an honest-but-curious adversary. This means that one needs to find three hosts who agree to set up an SMC server, and who are trustworthy enough not to deliberately break the computation, and clean the server from all intermediate data after the computation has finished (some challenges of real-world application deployment are described e.g. in [31]). Sacrificing in efficiency, we could use protocol sets like SPDZ [32] or MASCOT [33], which are actively secure against a dishonest majority. In any case, as far as we use secret sharing, we need to assume that at least one server should remain uncorrupted, or otherwise the computation parties will collaborate to reconstruct the clients’ secrets, regardless of the privacy-preserving algorithms used.

### Possible future work

As discussed e.g. in [9], the quasi-identifiers are often inconsistent in real applications, so exact comparison of values is not sufficient to achieve accurate linkage results, and some approximated string matching can be preferred. Our algorithms cannot be extended to this setting directly, since AES encryption maps similar strings to completely different values. We could take some ideas from some previous works, and hash all *n*-grams of a string instead of the entire string. Different *n*-grams should be encrypted with different AES keys to avoid leaking similarities in the string patterns. Nevertheless, this would already leak more information, and a corrupted server would be able to perform e.g. analysis of bigram frequencies, and there could possibly be more attacks inspired by [12], even though the attacks are adapted to Bloom filters that we do not use in our solution. We conclude that the extension to approximate matching would require a more careful design and security analysis.

## Conclusion

In this paper, we have proposed two methods for finding duplicates between several databases. The implementation has been benchmarked on data of 1000 medical centers with 10000 records each, giving quite positive results, which would be acceptable in real-world scenarios.

## Abbreviations

AES: Advanced encryption standard
CSV: Comma separated values
PPRL: Privacy-preserving record linkage
SMC: Secure multiparty computation
SHA: Secure hash algorithm
UOWHF: Universal one-way hash function
VM: Virtual machine
WAN: Wide area network
